# Outcome of Nonsurgical Management of Extra-Abdominal, Trunk, and Abdominal Wall Desmoid-Type Fibromatosis: A Population-Based Study in the Netherlands

**DOI:** 10.1155/2018/5982575

**Published:** 2018-06-21

**Authors:** Danique L. M. van Broekhoven, Arie J. Verschoor, Thijs van Dalen, Dirk J. Grünhagen, Michael A. den Bakker, Hans Gelderblom, Judith V. M. G. Bovee, Rick L. M. Haas, Han J. Bonenkamp, Frits van Coevorden, Diederik ten Oever, Winette T. A. van der Graaf, Uta E. Flucke, Elisabeth Pras, Anna K. L. Reyners, Anneke M. Westermann, Foppe Oldenburger, Cornelis Verhoef, Neeltje Steeghs

**Affiliations:** ^1^Department of Surgery, Erasmus MC Cancer Institute, P.O. Box 5201, 3008 AE Rotterdam, Netherlands; ^2^Department of Medical Oncology, Leiden University Medical Center, P.O. Box 9600, 2300 RC Leiden, Netherlands; ^3^Department of Surgery, University Medical Center Utrecht, P.O. Box 85500, 3508 GA Utrecht, Netherlands; ^4^Department of Surgery, Diakonessenhuis, Postbus 80250, 3508 TG Utrecht, Netherlands; ^5^Department of Pathology, Erasmus MC Cancer Institute, P.O. Box 5201, 3008 AE Rotterdam, Netherlands; ^6^Department of Pathology, Leiden University Medical Center, P.O. Box 9600, 2300 RC Leiden, Netherlands; ^7^Department of Radiotherapy, Netherlands Cancer Institute–Antoni van Leeuwenhoek, P.O. Box 90203, 1006 BE Amsterdam, Netherlands; ^8^Department of Surgery, Radboud University Medical Center, P.O. Box 9101, 6500 HB Nijmegen, Netherlands; ^9^Department of Surgery, Netherlands Cancer Institute–Antoni van Leeuwenhoek, P.O. Box 90203, 1006 BE Amsterdam, Netherlands; ^10^Department of Medical Oncology, Netherlands Cancer Institute–Antoni van Leeuwenhoek, P.O. Box 90203, 1006 BE Amsterdam, Netherlands; ^11^Deparment of Medical Oncology, Radboud University Medical Center, P.O. Box 9101, 6500 HB Nijmegen, Netherlands; ^12^Clinical and Translational Sarcoma Research, The Institute of Cancer Research, The Royal Marsden NHS Foundation Trust, 15 Cotswold Road, Sutton, London, Surrey SM2 5NG, UK; ^13^Department of Pathology, Radboud University Medical Center, P.O. Box 9101, 6500 HB Nijmegen, Netherlands; ^14^Department of Radiotherapy, University Medical Center Groningen, University of Groningen, P.O. Box 30001, 9700 RB Groningen, Netherlands; ^15^Department of Medical Oncology, University Medical Center Groningen, University of Groningen, P.O. Box 30001, 9700 RB Groningen, Netherlands; ^16^Department of Medical Oncology, Academic Medical Center, P.O. Box 22660, 1100 DD Amsterdam, Netherlands; ^17^Department of Radiotherapy, Academic Medical Center, P.O. Box 22660, 1100 DD Amsterdam, Netherlands

## Abstract

**Introduction:**

Nonsurgical management of patients with desmoid-type fibromatosis (DF) is increasing. This study tries to provide insight on type, usage, and outcome of first-line nonsurgical management strategies.

**Patients and Methods:**

From the Dutch Pathology Registry (PALGA), patients with extra-abdominal or trunk/abdominal wall DF, diagnosed between 1993 and 2013, were identified. First-line treatment was analyzed. Best response (BR) using RECIST criteria from start of treatment/surveillance until change of treatment or last follow-up was analyzed.

**Results:**

Ninety-one of the 1141 identified patients had first-line nonsurgical management. The percentage of patients treated nonsurgically increased from 0.6% in 1993–1998 to 12.8% in 2009–2013. Thirty-seven patients had surveillance (41%), 35 radiotherapy (38%), and 19 systemic treatment (21%). BR for surveillance was complete response (CR) in 2/37, partial response (PR) in 4/37, stable disease (SD) in 21/37, progressive disease (PD) in 5/37, and unknown in 5/37 patients. BR for radiotherapy was CR in 4/35, PR in 11/35, SD in 16/35, and unknown in 4/35. BR for systemic treatment was CR in 1/19, PR in 1/19, SD in 10/19, PD in 2/19, and unknown in 5/19. Totally, 91% of patients did not progress.

**Discussion:**

Given the low percentage (9%) of PD of nonsurgical management, these data can be used in shared decision making with the patient regarding optimal treatment.

## 1. Introduction

Desmoid-type fibromatosis (DF or aggressive fibromatosis) is an intermediate grade soft tissue tumor that does not metastasize, but can be locally aggressive [1]. For long, surgery has been the primary treatment for resectable tumors, with or without additional radiotherapy. Currently, a more conservative approach is applied based on reports of disease stabilization and spontaneous regression, and on documented progression after surgery as radical resection may be difficult to achieve [2, 3]. An epidemiological study conducted in extra-abdominal and trunk/abdominal wall DF patients in the Netherlands reported an increase in the use of nonsurgical modalities over the past decade [4].

A European consensus on the management of DF has recently been published, advocating active surveillance as the initial treatment modality, with systemic treatment, surgery or radiotherapy in case of tumor progression [5]. Despite a trend towards conservative treatment, knowledge on the outcome of different management modalities as first-line treatment is limited.

Studies on radiotherapy have described disease stabilization and tumor regression [6–8]. The literature on systemic treatment is limited, with a variety of treatment regimes, often applied at different stages of disease presentation [9–18]. Active surveillance is currently being investigated in a prospective setting by three different groups; a French group (NCT01801176), an Italian group (NCT02547831), and a Dutch group (NTR4714) [19]. Nonsurgical management of patients with DF is increasing. Population-based studies are needed to gain insight into the actual implementation of nonsurgical treatment in daily practice. This retrospective study provides insight into the application and outcome of all first-line treatment modalities in a nationwide cohort of DF patients during routine clinical care.

## 2. Patients and Methods

From the PALGA, the nationwide network and registry of histopathology and cytopathology in the Netherlands, patients diagnosed between 1-1-1993 and 31-12-2013 having extra-abdominal or trunk/abdominal wall DF were identified. The PALGA database contains encoded excerpts of all nationwide pathology examinations obtained by diagnostic procedure, including tissue biopsy or resection, since 1971 in selected laboratories and expanded to nationwide inclusion in 1991 [20]. Due to incomplete data registration, patients with disease presentation before 1993 were excluded. Excerpts contained standardized information: an encrypted patient identification, date of pathology report, age and gender of the patient, and the conclusion of the pathology reports. Reports were scored as biopsy, resection, or re-resection. Patients with diagnostic biopsy of DF without excision specimens within 6 months of biopsy were selected. Patients with excision specimens within 6 months of biopsy were considered to have initial surgical treatment. Exclusion criteria were intra-abdominal DF, recurrent disease at presentation, uncertain diagnosis, and initial surgical treatment.

Hospitals with more than 10 patients were contacted for information. Data collection was performed in seven tertiary referral centers, as most patients were referred to these centers after diagnosis. In addition to the PALGA database, center-based registrations were searched for patients. For all selected patients in these seven centers medical records were reviewed. From the excerpts and the medical records, data were collected on age, gender, year of diagnosis, localization, size, nuclear beta-catenin, *CTNNB1* mutations, *APC* mutations, treatment modalities, date of start of treatment, response to treatment, and toxicities. Only the first-line of treatment was documented.

Tumor localization was categorized as head/neck, trunk (including thoracic wall, breast, and back), abdominal wall, extremity, or groin. Type of systemic treatment was categorized as nonsteroidal anti-inflammatory drug (NSAID), antihormonal (HT), chemotherapy (ChT), or tyrosine kinase inhibitors (TKI).

Reports from all available imaging studies were reviewed. Best response to treatment was classified using RECIST 1.1 as complete response (CR), partial response (PR) in case of ≥30% decrease of the largest diameter , stable disease (SD), or progressive disease (PD) in case of ≥20% increase of the largest diameter based on reported measurements [21]. Date of the start of treatment was defined as the date of visit with the physician in which the treatment modality was initiated or date of start of radiotherapy. In most patients, active surveillance was initiated within 3 weeks after diagnosis. Results are shown as best response and time to progression (TTP). TTP was defined as the period from start of treatment to radiological PD as classified by RECIST 1.1. Follow-up period for each treatment was documented as time of start treatment or active surveillance until change of treatment or last documented follow-up visit, whichever came first.

Late toxicity after radiotherapy was retrospectively scored using RTOG-EORTC criteria [22].

Statistical analysis was performed using IBM SPSS Statistics 21. Continuous variables are shown as median with interquartile range (IQR), and categorical variables as numbers with percentages.

## 3. Results

**Figure 1:**
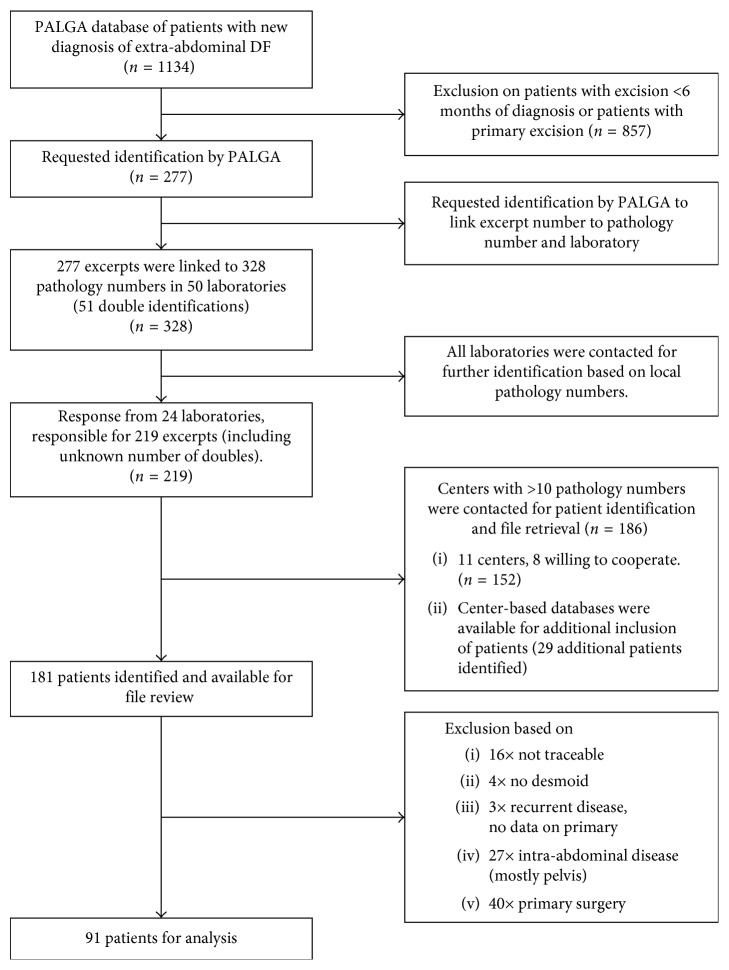
CONSORT diagram of patient selection.

The PALGA search covering the period between 1-1-1993 and 31-12-2013, identified 1134 patients with extra-abdominal and trunk/abdominal wall DF. Patients were selected using inclusion and exclusion criteria (Figure 1). Of these 1134 patients, 277 fulfilled the inclusion criteria for our study and 181 of these patients were treated in one of the seven hospitals selected for our study. Their files were reviewed for details on tumor characteristics and treatment modalities. After chart review, 90 additional patients were excluded because the chart review revealed additional information not available in the pathology report. Centre-based registrations provided data on additional patients (diagnosed in 2014). In total, 91 patients were included for further analysis based on inclusion and exclusion criteria. Baseline characteristics are listed in Table 1. Details on beta-catenin (*CTNNB1*) and *APC* gene mutation status were reported sporadically. To our knowledge, 6 patients with *APC* gene mutation were included. Due to the scarce data, these factors were not included in further analyses.

Based on initial management, patients were divided in 3 groups: active surveillance, radiotherapy, and systemic treatment. Outcomes for each group are listed in Table 2. Median follow-up for active surveillance, radiotherapy, and systemic treatment was 16 months (IQR 7–31), 44 months (IQR 24–62), and 5 months (IQR 2–12), respectively.

There is a clear increase in the use of nonsurgical management over the years, from 0.6% in 1993–1998 up to 12.8% in 2009–2013 (Table 3). Table 3 also presents data of 7 additional patients diagnosed in 2014, which were found during chart review.

### 3.1. Active Surveillance

Thirty-seven patients had active surveillance after diagnosis. Tumor localization was as follows: 3 patients with head/neck tumors, 13 patients with truncal tumors, 17 patients with abdominal wall tumors, and 4 patients with extremity tumors.

Best response during that period was spontaneous CR for 2 patients (5%), PR for 4 patients (11%), SD for 21 patients (57%), and PD for 5 patients (14%). For 5 patients, images required for RECIST were not available. CR was documented after 12 and 17 months, and PR was documented after 5, 10, 12, and 36 months. During the follow-up period, 13 patients had progressive disease with a median TTP of 7.3 months (IQR 4.1–11.9). In total, 22 patients (63%) were still under active surveillance at the date of last follow-up after a median of 16 months, including all patients with CR or PR (median duration of active surveillance for patients with CR and PR was 22 months; IQR 13–46). Of the 21 patients with SD as best outcome, 3 ended active surveillance due to complaints related to the tumor without actual progression and 5 patients ended due to progression (<20%). Thirteen patients with SD continued active surveillance till end of follow-up. Of the 5 patients with PD, 1 patient continued active surveillance. Two patients with unknown outcome continued active surveillance till end of follow-up.

### 3.2. Radiotherapy

Initial treatment was radiotherapy for 35 patients. Tumor localization was categorized as follows: 6 patients with head/neck tumors, 13 patients with truncal tumors, 1 patient with abdominal wall tumor, and 15 patients with extremity tumors.

Most patients (*n*=34) received 56 Gy in 28 fractions of 2 Gy or 25 fractions of 2 Gy and 2 fractions with 3 Gy. One patient with a tumor on the head/neck received 54 Gy over 30 sessions of 1.8 Gy.

Ten patients had no toxicity, 11 patients had grade 1 (mild joint stiffness, slight atrophy, and pigmentation change), 10 patients had grade 2 (patch atrophy, moderate fibrosis, and moderate joint stiffness), and one patient had grade 3 toxicity (severe joint stiffness). For three patients, insufficient data were available.

Best response to radiotherapy was CR in 4 patients (11%), PR in 11 patients (31%), and SD in 16 patients (46%). For 4 patients, no images were available to determine outcome according to RECIST. CR was documented after 12, 17, 26, and 29 months, and PR was documented after median 15.5 months (range 4–56 months). During follow-up, 2 patients developed PD with TTP of 31 and 47 months.

### 3.3. Systemic Treatment

Nineteen patients received initial systemic treatment. This consisted of nonsteroid anti-inflammatory drugs (NSAID) in 10 patients, antihormonal therapy (HT) in 5 patients, chemotherapy (ChT) in 1 patient, a tyrosine kinase inhibitor (TKI) in 1 patient, and a combination of HT and TKI in 1 patient. Details were missing for 1 patient.

Tumor localization was categorized as follows: thoracic/back in 9 patients, abdominal wall in 7 patients, extremity in 2 patients, and groin in 1 patient.

Best response during initial systemic treatment was CR for 1 patient (5%), PR for 1 patient (5%), SD for 10 patients (53%), PD for 2 patients (11%), and unknown for 5 patients (26%). CR was documented after 12 months, and PR was documented after 24 months. The female patient with CR received HT. The patient with PR received an NSAID. The 10 patients with SD were on NSAIDs (*n*=7), HT (*n*=2), and TKI (*n*=1). PD was seen after an NSAID (*n*=1) and ChT (*n*=1). During follow-up, 3 patients developed PD with TTP of 6.3, 7.1, and 7.2 months.

After initial systemic treatment, multiple systemic treatments were given to 10 patients in different regimens. Seven patients received 2 treatment regimens and three patients received a total of 4 treatment regimens.

## 4. Discussion

The change in treatment strategies from initial surgery with or without radiotherapy to initial nonsurgical management has been fueled by several studies and increasing expertise about this disease with its unpredictable behavior. The level of evidence is limited by the rarity of this disease. The Dutch cohort represents a unique and large group of patients with data on real-life practice. Within this group, analyses show that a 25% response rate and 52% stable disease rate was achieved using initial nonsurgical management.

Over the past 20 years, first-line nonsurgical management has increased up to 12.8%. Although the ratio between the time periods might be biased by several factors (such as limited numbers and registration), the trend towards nonsurgical management is evident and is expected to increase, as more specialists adhere to the current guidelines. Although there is an increase in nonsurgical management, still most patients are managed by surgery. Complaints such as pain or cosmetic reasons are reasons to do a resection. A resection could also have been performed for diagnostic purposes. Finally, limited experience with nonsurgical treatment in nonreferral centers could explain this high incidence of surgical excisions. As only chart review was done for patients with first-line nonsurgical treatment, we can only hypothesize about the reason for surgical management.

The literature on first-line nonsurgical management is limited, and most studies are reports from specialized centers. Retrospective studies with combined data from the French and Italian research groups reported promising results for all tumor localizations [23–25]. The present study was designed to provide more insight in common practice for this rare disease on a population-based level. In a national database of 1134 patients, the number of nonsurgically treated patients is small, but definitely increasing. Obviously, surgery has remained the first-line treatment over the last 20 years, but a paradigm shift towards active surveillance can be observed. The surveillance cohort is the largest group among patients managed nonsurgically. Radiotherapy was the second used treatment modality. In general, radiotherapy is indicated only in serious cases where progression of the tumor can lead to serious morbidity [6]. The risk of acute and late toxicity, including secondary malignancy, restricts its application, particularly in the young age group and in those patients with abdominal locations. Compared to the study by Colombo et al., the current study showed a high frequency (38%) of patients treated with radiotherapy compared to 3% in the French/Italian study [23]. No other studies are available, and the reason for this high number of primary irradiated tumors is unknown. Compared to the surveillance cohort, there are a relative high number of patients with an extremity localization in the radiotherapy group. Although we do not exactly know, it could be that patients with an extremity tumor are less likely to be referred, and when they are referred, they are symptomatic and therefore prone to have surgery or radiotherapy. The small numbers of patients who received systemic treatment reflect the limited evidence for any of the treatment options and lack of clinical studies in the Netherlands. This study was not designed to compare the outcome of the different treatment modalities, merely to report common practice over the years.

Overall, outcome of first-line nonsurgical treatment was good with a 25% response rate and 52% stable disease rate. Of all evaluable patients, 90% did not have early progression of disease. Among patients under active surveillance, 16% showed spontaneous regression and 57% disease stabilization. These results might be biased because in many cases, choice for first-line treatment was made after referring the patient to a tertiary referral center which enabled the physicians to observe the natural behavior of the tumor, thereby selecting patients for either active surveillance or more aggressive treatments. Referring these patients to a tertiary referral center is common practice in the Netherlands, and so this reflects the common practice in the Netherlands. For radiotherapy, the patients in the present study received radiotherapy at the recommended dose of 50–56 Gy [6–8, 26]. Results of radiotherapy showed a response in 43% and SD in 46% of the patients. During the follow-up period (median of 44 months (IQR 24–62)), only 2 patients had disease progression with long TTPs of 31 and 47 months. These results are promising and might seem to advocate radiotherapy. However, radiotherapy might be considered an aggressive treatment for this intermediate grade tumor, usually reserved for patients with advanced disease. Especially in younger patients, given the low, but present long-term risk on irradiation-induced sarcomas, radiotherapy is not deemed as first-line treatment. When systemic treatment is chosen, a large variety of possible agents and regimens are applied (despite the lack of a specific registration for DF), such as hormonal agents, NSAIDs, chemotherapy, and angiogenesis inhibitors, making comparison impossible. Although the group in the present study was small and diverse, results show stabilization and response in 63% of patients. Again, due to the large variety, no conclusions can be made for on preference of specific agents or regimens.

Given the lack of randomized studies, treatment decisions should be made during multidisciplinary expert meetings. Decision making should take into account location and growth of the tumor, but in particularly the symptoms of the patient. A recent study by the French patients advocacy group SOS desmoid showed that 63% of patients that participated in a survey reported pain [27].

The optimal first-line nonsurgical management of DF has been discussed by many groups, predominantly based on expert opinions and specific treatment modalities. The European consensus, reported by Kasper et al. [5], advises to start with active surveillance and switch to active treatment in case of 3 subsequent reports of progression and that treatment should be guided by tumor localization. There is no staging system available to predict outcome at the time of diagnosis. Predictive factors have been described, such as age, tumor localization, and *CTNNB1* mutations [28–33]. Recent data on *CTNNB1* mutations show different behavior for tumors with different mutations. In the future, these mutations could play an important role when deciding to initiate specific treatment modalities. Moreover, it is increasingly important to recognize the lack of association between radiological volume and symptoms [34–36]. Given the chronic condition and the spontaneous fluctuations of the disease, this should be taken into account in any decision that will be taken.

By the use of PALGA, the Dutch pathology registry, and the long study period, we have tried to be as inclusive as possible. Because referral for a desmoid-type fibromatosis to one of the sarcoma referral centers is standard practice in the Netherlands, we consider this overview as unbiased. However, a part of the patients identified from PALGA were not included because they were treated outside the referral centers. The referral of these patients is essential to develop expertise in the treatment of this rare disease.

A limitation of the study is its retrospective nature. As a result, details on symptoms during or after treatment are lacking, which could have provided insight into the way decisions to either management had been taken. Therefore, no comparisons can be made between the different strategies. The natural behavior of these tumors is variable, varying from spontaneous regression to long-term disease stabilization and rapid progression. In the absence of randomization, no clear recommendations can be given.

## 5. Conclusion

Desmoid-type fibromatosis remains a rare disease, for which several treatment modalities are available. Active surveillance seems to be a good and safe initial treatment, with options for active treatment in case of progression. Importantly, expected benefits from therapy should be well balanced against potential treatment-induced chronic and late effects.

## Figures and Tables

**Table 1:** Baseline characteristics.

	All patients		Active Surveillance		Radiotherapy		Systemic treatment	
	*N*	%	*N*	%	*N*	%	*N*	%
*Gender*								
Male	30	33	9	24.3	12	34.3	9	47.4
Female	61	67	28	75.7	23	65.7	10	52.6

*Age (years)*								
Median (IQR)	39 (33.1–52.2)		36 (31.2–51.6)		43.6 (39.4–52.4)		34.8 (23.3–46.3)	

*Localization*								
Head/neck	9	9.9	3	8.1	6	17.1	—	—
Thorax/back	35	38.5	13	35.1	13	37.1	9	47.4
Abdominal wall	25	27.5	17	45.9	1	2.9	7	36.8
Extremity	21	23.1	4	10.8	15	42.9	2	10.5
Others^*∗*^	1	1.1	—	—	—	—	1	5.3

*Size*								
<5 cm	25	27.5	16	43.2	7	20.0	2	10.5
5–10 cm	48	52.7	18	48.6	19	54.3	11	57.9
>10 cm	15	16.5	2	5.4	8	22.9	5	26.3
Missing data	3	3.3	1	2.7	1	2.9	1	5.3

*Beta-catenin (nuclear)*								
Positive	56	61.5	28	75.7	16	45.7	12	63.2
Negative	10	11	3	8.1	6	17.1	1	5.3
Unknown	25	27.5	6	16.2	13	37.1	6	31.6

*N* = number of patients; cm = centimeter; IQR = interquartile range; ^*∗*^groin.

**Table 2:** Outcome of nonsurgical treatment, using best response according to RECIST.

	CR		PR		SD		PD		Unknown		Total
	*N*	%	*N*	%	*N*	%	*N*	%	*N*	%	*N*
Active surveillance	2	5.4%	4	10.8%	21	56.8%	5	13.5%	5	13.5%	37
Radiotherapy	4	11.4%	11	31.4%	16	45.7%	0	0%	4	11.4%	35
Systemic treatment	1	5.3%	1	5.3%	10	52.6%	2	10.5%	5	26.3%	19

*N* = number of patients; CR = complete response; PR = partial response; SD = stable disease; PD = progressive disease.

**Table 3:** First-line nonsurgical treatment per 5-year time period.

	1993–1998	1999–2003	2004–2008	2009–2013	2014	Total
	*N*	*N*	*N*	*N*	*N*	*N*
PALGA registration [4]	180	185	331	438		1134
First-line treatment	1	5	22	56	7	91
Stratified treatment						
Active surveillance	0	0	5	26	6	37
Radiotherapy	0	1	13	20	1	35
Systemic treatment	1	4	4	10	0	19
Percentage^*∗*^	0.6%	2.7%	6.6%	12.8%		8.0%

*N* = number of patients. ^*∗*^Percentage of nonsurgical treatment compared to overall diagnoses as documented in the PALGA registration.

## Data Availability

Requests for the raw data will be considered by the corresponding author.
